# Supplementary material for: Gender variations in citation distributions in medicine are very small and due to self-citation and journal prestige
Source: eLife. 2019 Jul 15;8:e45374. doi: 10.7554/eLife.45374 (PMC6677534; doi:10.7554/eLife.45374)
Supplement: Figure 2—source data 1. [file elife-45374-fig2-data1.docx]

| **Figure 2-source data 1.** Tweedie regression results | | | | | | | | | | | | | | |
| --- | --- | --- | --- | --- | --- | --- | --- | --- | --- | --- | --- | --- | --- | --- |
|  | | | | | **Raw parameters** | | | | | **Standardized parameters** | | | | |
| **Outcome** | **Model** | **Predictor** | **Estimate** | **Std. Error** | | **EE** | **EE.LCL** | **EE.UCL** | **Estimate** | | **Std. Error** | **EE** | **EE.LCL** | **EE.UCL** |
| NCS | Sample 1 | (Intercept) | -0.39 | 0.0020 | | 0.68 | 0.68 | 0.68 | 0.03 | | 0.0010 | 1.03 | 1.03 | 1.03 |
| NCS | Sample 1 | case | -0.02 | 0.0020 | | 0.98 | 0.98 | 0.98 | -0.02 | | 0.0020 | 0.98 | 0.98 | 0.98 |
| NCS | Sample 1 | n_authors | -0.01 | 0.0002 | | 0.99 | 0.99 | 0.99 | -0.06 | | 0.0018 | 0.94 | 0.94 | 0.94 |
| NCS | Sample 1 | int_collab | 0.11 | 0.0024 | | 1.12 | 1.11 | 1.12 | 0.11 | | 0.0024 | 1.12 | 1.11 | 1.12 |
| NCS | Sample 1 | selfcit | 0.07 | 0.0001 | | 1.07 | 1.07 | 1.07 | 0.51 | | 0.0009 | 1.66 | 1.65 | 1.66 |
| NCS | Sample 1 | mncs_journal | 0.26 | 0.0006 | | 1.29 | 1.29 | 1.29 | 0.50 | | 0.0013 | 1.65 | 1.64 | 1.65 |
| NCS | Sample 2 | (Intercept) | -0.46 | 0.0025 | | 0.63 | 0.63 | 0.64 | 0.01 | | 0.0012 | 1.01 | 1.01 | 1.01 |
| NCS | Sample 2 | case | -0.01 | 0.0025 | | 0.99 | 0.98 | 0.99 | -0.01 | | 0.0025 | 0.99 | 0.98 | 0.99 |
| NCS | Sample 2 | n_authors | 0.00 | 0.0003 | | 1.00 | 1.00 | 1.00 | -0.03 | | 0.0023 | 0.97 | 0.97 | 0.98 |
| NCS | Sample 2 | int_collab | 0.10 | 0.0031 | | 1.11 | 1.10 | 1.11 | 0.10 | | 0.0031 | 1.11 | 1.10 | 1.11 |
| NCS | Sample 2 | selfcit | 0.07 | 0.0001 | | 1.07 | 1.07 | 1.07 | 0.46 | | 0.0008 | 1.58 | 1.58 | 1.59 |
| NCS | Sample 2 | mncs_journal | 0.30 | 0.0009 | | 1.35 | 1.34 | 1.35 | 0.57 | | 0.0016 | 1.76 | 1.75 | 1.77 |
| NCS | Sample 3 | (Intercept) | -0.36 | 0.0033 | | 0.70 | 0.69 | 0.70 | 0.03 | | 0.0016 | 1.03 | 1.02 | 1.03 |
| NCS | Sample 3 | case | -0.04 | 0.0033 | | 0.96 | 0.96 | 0.97 | -0.04 | | 0.0033 | 0.96 | 0.96 | 0.97 |
| NCS | Sample 3 | n_authors | 0.00 | 0.0004 | | 1.00 | 0.99 | 1.00 | -0.04 | | 0.0029 | 0.97 | 0.96 | 0.97 |
| NCS | Sample 3 | int_collab | 0.13 | 0.0039 | | 1.14 | 1.13 | 1.15 | 0.13 | | 0.0039 | 1.14 | 1.13 | 1.15 |
| NCS | Sample 3 | selfcit | 0.05 | 0.0001 | | 1.05 | 1.05 | 1.05 | 0.36 | | 0.0010 | 1.43 | 1.42 | 1.43 |
| NCS | Sample 3 | mncs_journal | 0.26 | 0.0010 | | 1.30 | 1.29 | 1.30 | 0.49 | | 0.0019 | 1.63 | 1.62 | 1.64 |
| Dispersion parameters: Sample 1= 1.002, Sample 2= 1.010, Sample 3= 1.003 | | | | | | |  |  |  | |  |  |  |  |
| *Note:* |  |  |  |  | |  |  |  |  | |  |  |  |  |
| EE : Exponentiated estimate | | | |  | |  |  |  |  | |  |  |  |  |
| EE.LCL : Lower confidence limit of exponentiated estimate | | | |  | |  |  |  |  | |  |  |  |  |
| EE.UCL : Upper confidence limit of exponentiated estimate | | | |  | |  |  |  |  | |  |  |  |  |
