# Supplementary material for: Gender variations in citation distributions in medicine are very small and due to self-citation and journal prestige
Source: eLife. 2019 Jul 15;8:e45374. doi: 10.7554/eLife.45374 (PMC6677534; doi:10.7554/eLife.45374)
Supplement: Figure 2—source data 2. [file elife-45374-fig2-data2.docx]

| **Figure 2-source data 2.** Regression results for Tweedie regressions on the full, unmatched sample, using NCS as outcome. | | | | | | | |
| --- | --- | --- | --- | --- | --- | --- | --- |
| **Outcome** | **Model** | **Predictor** | **Estimate** | **Std. Error** | **EE** | **EE.LCL** | **EE.UCL** |
| NCS | F_First | (Intercept) | 0.00 | 0.0013 | 1.00 | 1.00 | 1.01 |
| NCS | F_First | f_first | -0.01 | 0.0018 | 0.99 | 0.98 | 0.99 |
| NCS | F_First | n_authors | -0.02 | 0.0016 | 0.98 | 0.98 | 0.98 |
| NCS | F_First | int_collab | 0.12 | 0.0022 | 1.13 | 1.13 | 1.14 |
| NCS | F_First | selfcit | 0.42 | 0.0005 | 1.52 | 1.51 | 1.52 |
| NCS | F_First | mncs_journal | 0.54 | 0.0011 | 1.71 | 1.71 | 1.72 |
| NCS | F_Last | (Intercept) | 0.00 | 0.0012 | 1.00 | 1.00 | 1.00 |
| NCS | F_Last | f_last | -0.01 | 0.0020 | 0.99 | 0.99 | 1.00 |
| NCS | F_Last | n_authors | -0.02 | 0.0016 | 0.98 | 0.98 | 0.98 |
| NCS | F_Last | int_collab | 0.12 | 0.0022 | 1.13 | 1.13 | 1.14 |
| NCS | F_Last | selfcit | 0.42 | 0.0005 | 1.52 | 1.51 | 1.52 |
| NCS | F_Last | mncs_journal | 0.54 | 0.0011 | 1.71 | 1.71 | 1.72 |
| NCS | F_Both | (Intercept) | 0.00 | 0.0011 | 1.00 | 1.00 | 1.00 |
| NCS | F_Both | f_both | -0.02 | 0.0025 | 0.98 | 0.97 | 0.98 |
| NCS | F_Both | n_authors | -0.02 | 0.0016 | 0.98 | 0.98 | 0.98 |
| NCS | F_Both | int_collab | 0.12 | 0.0022 | 1.13 | 1.13 | 1.14 |
| NCS | F_Both | selfcit | 0.42 | 0.0005 | 1.52 | 1.51 | 1.52 |
| NCS | F_Both | mncs_journal | 0.54 | 0.0011 | 1.71 | 1.71 | 1.72 |
| Dispersion parameters: Sample 1= 1.027, Sample 2= 1.028, Sample 3= 1.027 | | |  |  |  |  |  |
| *Note:* |  |  |  |  |  |  |  |
| EE : Exponentiated estimate |  |  |  |  |  |  |  |
| EE.LCL : Lower confidence limit of exponentiated estimate |  |  |  |  |  |  |  |
| EE.UCL : Upper confidence limit of exponentiated estimate |  |  |  |  |  |  |  |
