# Supplementary material for: Gender variations in citation distributions in medicine are very small and due to self-citation and journal prestige
Source: eLife. 2019 Jul 15;8:e45374. doi: 10.7554/eLife.45374 (PMC6677534; doi:10.7554/eLife.45374)
Supplement: Figure 2—source data 3. [file elife-45374-fig2-data3.docx]

| **Figure 2-source data 3.** Regression results for the three negative binomial regressions with times cited (CS) as outcome. | | | | | | | |
| --- | --- | --- | --- | --- | --- | --- | --- |
| **Outcome** | **Model** | **Predictor** | **Estimate** | **Std. Error** | **IRR** | **IRR.LCL** | **IRR.UCL** |
| CS | Sample 1 | (Intercept) | 1.44 | 0.0019 | 4.22 | 4.21 | 4.24 |
| CS | Sample 1 | case | 0.00 | 0.0017 | 1.00 | 0.99 | 1.00 |
| CS | Sample 1 | n_authors | 0.00 | 0.0002 | 1.00 | 1.00 | 1.00 |
| CS | Sample 1 | int_collab | 0.04 | 0.0021 | 1.04 | 1.04 | 1.05 |
| CS | Sample 1 | selfcit | 0.14 | 0.0002 | 1.15 | 1.15 | 1.15 |
| CS | Sample 1 | mncs_journal | 0.43 | 0.0009 | 1.53 | 1.53 | 1.54 |
| CS | Sample 2 | (Intercept) | 1.42 | 0.0024 | 4.13 | 4.11 | 4.15 |
| CS | Sample 2 | case | -0.01 | 0.0021 | 0.99 | 0.98 | 0.99 |
| CS | Sample 2 | n_authors | 0.00 | 0.0003 | 1.00 | 1.00 | 1.00 |
| CS | Sample 2 | int_collab | 0.04 | 0.0027 | 1.05 | 1.04 | 1.05 |
| CS | Sample 2 | selfcit | 0.14 | 0.0003 | 1.15 | 1.15 | 1.15 |
| CS | Sample 2 | mncs_journal | 0.44 | 0.0011 | 1.55 | 1.55 | 1.56 |
| CS | Sample 3 | (Intercept) | 1.41 | 0.0032 | 4.11 | 4.08 | 4.14 |
| CS | Sample 3 | case | -0.01 | 0.0028 | 0.99 | 0.98 | 0.99 |
| CS | Sample 3 | n_authors | 0.00 | 0.0004 | 1.00 | 1.00 | 1.00 |
| CS | Sample 3 | int_collab | 0.04 | 0.0035 | 1.04 | 1.03 | 1.05 |
| CS | Sample 3 | selfcit | 0.14 | 0.0004 | 1.15 | 1.15 | 1.15 |
| CS | Sample 3 | mncs_journal | 0.44 | 0.0015 | 1.55 | 1.55 | 1.56 |
| Dispersion parameters: Sample 1= 1.000, Sample 2= 1.000, Sample 3= 1.000 | | |  |  |  |  |  |
| *Note:* |  |  |  |  |  |  |  |
| IRR : Incidence rate ratios |  |  |  |  |  |  |  |
| IRR.LCL : Lower confidence limit of incidence rate ratios |  |  |  |  |  |  |  |
| IRR.UCL : Upper confidence limit of incidence rate ratios |  |  |  |  |  |  |  |
