# Supplementary material for: Gender variations in citation distributions in medicine are very small and due to self-citation and journal prestige
Source: eLife. 2019 Jul 15;8:e45374. doi: 10.7554/eLife.45374 (PMC6677534; doi:10.7554/eLife.45374)
Supplement: Figure 2—source data 4. [file elife-45374-fig2-data4.docx]

| **Figure 2-source data 4.** Tweedie regression of standardized parameters, using MNCS Journal quantiles rather than measurements. | | | | | | | |
| --- | --- | --- | --- | --- | --- | --- | --- |
| **Outcome** | **Model** | **Predictor** | **Estimate** | **Std. Error** | **EE** | **EE.LCL** | **EE.UCL** |
| NCS | Sample 1 | (Intercept) | -0.32 | 0.0019 | 0.73 | 0.72 | 0.73 |
| NCS | Sample 1 | case | -0.02 | 0.0020 | 0.98 | 0.98 | 0.99 |
| NCS | Sample 1 | n_authors | -0.07 | 0.0019 | 0.93 | 0.93 | 0.93 |
| NCS | Sample 1 | int_collab | 0.08 | 0.0025 | 1.08 | 1.08 | 1.09 |
| NCS | Sample 1 | selfcit | 0.54 | 0.0009 | 1.71 | 1.71 | 1.72 |
| NCS | Sample 1 | mncs_j_high | 1.19 | 0.0039 | 3.28 | 3.25 | 3.30 |
| NCS | Sample 1 | mncs_j_med | 0.56 | 0.0021 | 1.75 | 1.74 | 1.75 |
| NCS | Sample 2 | (Intercept) | -0.34 | 0.0024 | 0.71 | 0.71 | 0.72 |
| NCS | Sample 2 | case | -0.01 | 0.0026 | 0.99 | 0.99 | 1.00 |
| NCS | Sample 2 | n_authors | -0.03 | 0.0024 | 0.97 | 0.96 | 0.97 |
| NCS | Sample 2 | int_collab | 0.08 | 0.0032 | 1.08 | 1.08 | 1.09 |
| NCS | Sample 2 | selfcit | 0.47 | 0.0008 | 1.61 | 1.60 | 1.61 |
| NCS | Sample 2 | mncs_j_high | 1.23 | 0.0051 | 3.41 | 3.37 | 3.44 |
| NCS | Sample 2 | mncs_j_med | 0.57 | 0.0027 | 1.76 | 1.75 | 1.77 |
| NCS | Sample 3 | (Intercept) | -0.34 | 0.0033 | 0.72 | 0.71 | 0.72 |
| NCS | Sample 3 | case | -0.03 | 0.0035 | 0.97 | 0.96 | 0.98 |
| NCS | Sample 3 | n_authors | -0.06 | 0.0032 | 0.94 | 0.93 | 0.95 |
| NCS | Sample 3 | int_collab | 0.09 | 0.0042 | 1.09 | 1.08 | 1.10 |
| NCS | Sample 3 | selfcit | 0.40 | 0.0011 | 1.50 | 1.49 | 1.50 |
| NCS | Sample 3 | mncs_j_high | 1.26 | 0.0065 | 3.53 | 3.48 | 3.57 |
| NCS | Sample 3 | mncs_j_med | 0.58 | 0.0037 | 1.78 | 1.77 | 1.79 |
| Dispersion parameters: Sample 1= 1.058, Sample 2= 1.098, Sample 3= 1.137 | | |  |  |  |  |  |
| *Note:* |  |  |  |  |  |  |  |
| EE : Exponentiated estimate |  |  |  |  |  |  |  |
| EE.LCL : Lower confidence limit of exponentiated estimate |  |  |  |  |  |  |  |
| EE.UCL : Upper confidence limit of exponentiated estimate |  |  |  |  |  |  |  |
| mncs_j_high : MNCS Journal scores in and above the 95th percentile |  |  |  |  |  |  |  |
| mncs_j_med: MNCS Journal scores from the 50th to the 94th percentile |  |  |  |  |  |  |  |
