# Supplementary material for: Gender variations in citation distributions in medicine are very small and due to self-citation and journal prestige
Source: eLife. 2019 Jul 15;8:e45374. doi: 10.7554/eLife.45374 (PMC6677534; doi:10.7554/eLife.45374)
Supplement: Figure 4—source data 1. [file elife-45374-fig4-data1.docx]

| **Figure 4-source data 1.** Logistic regression results | | | | | | | | | | | | |
| --- | --- | --- | --- | --- | --- | --- | --- | --- | --- | --- | --- | --- |
|  | | | **Raw parameters** | | | | | **Standardized parameters** | | | | |
| **Outcome** | **Model** | **Predictor** | **Estimate** | **Std. Error** | **OR** | **OR.LCL** | **OR.UCL** | **Estimate** | **Std. Error** | **OR** | **OR.LCL** | **OR.UCL** |
| case | Sample 1 | (Intercept) | 0.00 | 0.0041 | 1.00 | 0.99 | 1.01 | 0.00 | 0.0020 | 1.00 | 1.00 | 1.00 |
| case | Sample 1 | n_authors | 0.02 | 0.0006 | 1.02 | 1.01 | 1.02 | 0.12 | 0.0044 | 1.13 | 1.12 | 1.14 |
| case | Sample 1 | int_collab | -0.03 | 0.0051 | 0.97 | 0.96 | 0.98 | -0.03 | 0.0051 | 0.97 | 0.96 | 0.98 |
| case | Sample 1 | selfcit | -0.02 | 0.0007 | 0.98 | 0.98 | 0.98 | -0.15 | 0.0047 | 0.86 | 0.86 | 0.87 |
| case | Sample 1 | mncs_journal | -0.03 | 0.0023 | 0.97 | 0.96 | 0.97 | -0.07 | 0.0044 | 0.94 | 0.93 | 0.94 |
| case | Sample 2 | (Intercept) | 0.12 | 0.0051 | 1.13 | 1.12 | 1.14 | 0.00 | 0.0025 | 1.00 | 0.99 | 1.00 |
| case | Sample 2 | n_authors | -0.01 | 0.0007 | 0.99 | 0.99 | 0.99 | -0.08 | 0.0055 | 0.93 | 0.92 | 0.94 |
| case | Sample 2 | int_collab | -0.01 | 0.0064 | 0.99 | 0.98 | 1.01 | -0.01 | 0.0064 | 0.99 | 0.98 | 1.01 |
| case | Sample 2 | selfcit | -0.01 | 0.0008 | 0.99 | 0.98 | 0.99 | -0.10 | 0.0059 | 0.91 | 0.90 | 0.92 |
| case | Sample 2 | mncs_journal | -0.03 | 0.0029 | 0.97 | 0.96 | 0.98 | -0.06 | 0.0055 | 0.94 | 0.93 | 0.95 |
| case | Sample 3 | (Intercept) | 0.14 | 0.0069 | 1.15 | 1.13 | 1.16 | 0.00 | 0.0033 | 1.00 | 0.99 | 1.01 |
| case | Sample 3 | n_authors | 0.00 | 0.0009 | 1.00 | 1.00 | 1.00 | -0.02 | 0.0073 | 0.98 | 0.97 | 1.00 |
| case | Sample 3 | int_collab | 0.01 | 0.0083 | 1.01 | 0.99 | 1.03 | 0.01 | 0.0083 | 1.01 | 0.99 | 1.03 |
| case | Sample 3 | selfcit | -0.03 | 0.0012 | 0.97 | 0.97 | 0.97 | -0.21 | 0.0082 | 0.81 | 0.80 | 0.82 |
| case | Sample 3 | mncs_journal | -0.06 | 0.0040 | 0.94 | 0.94 | 0.95 | -0.11 | 0.0076 | 0.90 | 0.88 | 0.91 |
| Dispersion parameters: Sample 1=1.000, Sample 2= 1.000, Sample 3= 1.000 | | | | |  |  |  |  |  |  |  |  |
| *Note:* |  |  |  |  |  |  |  |  |  |  |  |  |
| OR : Odds ratios |  |  |  |  |  |  |  |  |  |  |  |  |
| OR.LCL : Lower confidence limit of odds ratios | | | |  |  |  |  |  |  |  |  |  |
| OR.UCL : Upper confidence limit of odds ratios | | |  |  |  |  |  |  |  |  |  |  |
