# Supplementary material for: Gender variations in citation distributions in medicine are very small and due to self-citation and journal prestige
Source: eLife. 2019 Jul 15;8:e45374. doi: 10.7554/eLife.45374 (PMC6677534; doi:10.7554/eLife.45374)
Supplement: Figure 7—source data 1. [file elife-45374-fig7-data1.docx]

| **Figure 7-source data 1.** Excluded countries due to unreliable gender assignments from first name. | |
| --- | --- |
| **Country** | **Reliability** |
| Taiwan | 0.29 |
| Vietnam | 0.35 |
| China | 0.38 |
| Mongolia | 0.49 |
| Myanmar [Burma] | 0.56 |
| Singapore | 0.66 |
| South Korea | 0.71 |
| Malaysia | 0.73 |
| Cambodia | 0.74 |
| Laos | 0.78 |
| Fiji | 0.79 |
| Brunei | 0.81 |
| Sri Lanka | 0.83 |
| Swaziland | 0.83 |
| Botswana | 0.83 |
| Seychelles | 0.86 |
| Zimbabwe | 0.87 |
| Nigeria | 0.88 |
| Indonesia | 0.88 |
| Burundi | 0.89 |
| Madagascar | 0.89 |
| Zambia | 0.90 |
| Guyana | 0.90 |
