# Supplementary material for: Gender variations in citation distributions in medicine are very small and due to self-citation and journal prestige
Source: eLife. 2019 Jul 15;8:e45374. doi: 10.7554/eLife.45374 (PMC6677534; doi:10.7554/eLife.45374)
Supplement: Figure 7—source data 2. [file elife-45374-fig7-data2.docx]

**Specialty disambiguation**

To adjust for medical specialties, papers need to be classified, preferably as one specialty per paper. Such a classification is not readily available in PubMed nor Web of Science. Darmoni et al. (Darmoni et al., 2006) designed an algorithm allowing such classifications from MeSH terms assigned to papers. The algorithm operates on a MeSH-specialty assignment table (Darmoni et al., 2006; Gehanno et al., 2011) which is available through an API at <http://www.hetop.eu>. For each MeSH term assigned to a paper, the corresponding specialties are counted, so that the paper is classified by the most common specialty. The counts are weighted by whether the MeSH term is a major index term (full weight) or not (half weight). The list of specialties is more detailed than most other such lists, containing a total of 124 specialties. We have summarised this list into five main specialties, based on the expert field knowledge of R.J. These assignments are also available from supplementary Table S11.

| **Figure 7-source data 2.** List of specialty and main specialty designation, and number of papers per specialty for the full sample. | | |
| --- | --- | --- |
| **Specialty** | **Main specialty** | **Number of papers** |
| environment and public health | basic science | 63147 |
| epidemiology | basic science | 58666 |
| cytology | basic science | 53840 |
| physiology | basic science | 44743 |
| virology | basic science | 34987 |
| bacteriology | basic science | 32552 |
| information science | basic science | 13815 |
| nutrition | basic science | 6058 |
| mycology | basic science | 4488 |
| toxicology | basic science | 4326 |
| equipment and supplies | basic science | 4209 |
| parasitology | basic science | 3700 |
| pharmacy | basic science | 3334 |
| economics | basic science | 3249 |
| education | basic science | 2437 |
| histology | basic science | 1867 |
| environnement | basic science | 1599 |
| organization and administration | basic science | 1261 |
| evidence-based medicine | basic science | 891 |
| statistics | basic science | 861 |
| research | basic science | 845 |
| phytotherapy | basic science | 655 |
| embryology | basic science | 618 |
| microbiology | basic science | 584 |
| history of medicine | basic science | 411 |
| law | basic science | 270 |
| medical informatics | basic science | 265 |
| disease transmission | basic science | 224 |
| social affairs | basic science | 195 |
| pharmacology | basic science | 162 |
| biochemistry | basic science | 143 |
| ethics | basic science | 127 |
| developmental biology and medicine | basic science | 109 |
| anatomy | basic science | 31 |
| biomedical engineering | basic science | 27 |
| molecular biology | basic science | 20 |
| biophysics | basic science | 9 |
| neurology | hospital based | 94856 |
| psychiatry | hospital based | 19820 |
| dermatology | hospital based | 6820 |
| veterinary medicine | hospital based | 5179 |
| addictology | hospital based | 4301 |
| diagnostic imaging | hospital based | 2495 |
| nursing care | hospital based | 1033 |
| anesthesiology | hospital based | 959 |
| emergency medicine | hospital based | 880 |
| risk management | hospital based | 216 |
| nuclear medicine | hospital based | 189 |
| forensic medicine | hospital based | 146 |
| pathology | hospital based | 38 |
| oncology | medical | 133393 |
| cardiology | medical | 92712 |
| genetics | medical | 72929 |
| allergy and immunology | medical | 50977 |
| rheumatology | medical | 43453 |
| drugs | medical | 43306 |
| diagnosis | medical | 39095 |
| gastroenterology | medical | 28805 |
| therapeutics | medical | 24065 |
| pulmonary disease | medical | 21577 |
| endocrinology | medical | 18631 |
| hematology | medical | 17051 |
| infectious disease medicine | medical | 16778 |
| metabolism | medical | 11127 |
| geriatrics | medical | 7902 |
| nephrology | medical | 4809 |
| hepatology | medical | 3411 |
| occupational medicine | medical | 2375 |
| pain | medical | 1719 |
| preventive medicine | medical | 1089 |
| internal medicine | medical | 1080 |
| sleep medicine specialty | medical | 855 |
| military medicine | medical | 827 |
| disability | medical | 711 |
| palliative medicine | medical | 391 |
| physical medicine and rehabilitation | medical | 212 |
| ambulatory care | medical | 188 |
| humanitarian medicine | medical | 181 |
| critical care | medical | 139 |
| family medicine | medical | 136 |
| aerospace medicine | medical | 131 |
| alternative medicine | medical | 126 |
| prison medicine | medical | 89 |
| mountain medicine | medical | 60 |
| physiotherapy | medical | 60 |
| venereology | medical | 56 |
| travel medicine | medical | 54 |
| home care | medical | 40 |
| thermal medicine | medical | 16 |
| homeopathy | medical | 13 |
| hemobiology-blood transfusion | medical | 11 |
| naval medicine | medical | 5 |
| pediatrics | pediatric | 28814 |
| neonatology | pediatric | 1209 |
| foetology | pediatric | 1184 |
| adolescent medicine | pediatric | 834 |
| school medicine | pediatric | 62 |
| surgery | surgical/procedural | 30864 |
| ophthalmology | surgical/procedural | 22728 |
| urology | surgical/procedural | 15972 |
| dentistry | surgical/procedural | 13953 |
| gynecology | surgical/procedural | 8753 |
| traumatology | surgical/procedural | 6756 |
| otolaryngology | surgical/procedural | 6439 |
| vascular medicine and surgery | surgical/procedural | 3062 |
| reproductive medicine | surgical/procedural | 3033 |
| obstetrics | surgical/procedural | 2649 |
| mastology | surgical/procedural | 1912 |
| transplantation | surgical/procedural | 1693 |
| sports medicine | surgical/procedural | 1494 |
| podiatry | surgical/procedural | 452 |
| orthopedics | surgical/procedural | 253 |
| burns | surgical/procedural | 246 |
| oral medicine | surgical/procedural | 82 |
| periodontics | surgical/procedural | 60 |
| acupuncture | surgical/procedural | 57 |
| plastic and esthetic surgery | surgical/procedural | 46 |
| oral surgical procedures | surgical/procedural | 30 |
| thoracic and cardiovascular surgery | surgical/procedural | 16 |
| neurosurgery | surgical/procedural | 6 |
| orthodontics | surgical/procedural | 1 |
