# Supplementary material for: Gender variations in citation distributions in medicine are very small and due to self-citation and journal prestige
Source: eLife. 2019 Jul 15;8:e45374. doi: 10.7554/eLife.45374 (PMC6677534; doi:10.7554/eLife.45374)
Supplement: Figure 7—source data 3. [file elife-45374-fig7-data3.docx]

**Geographical regions**

| **Figure 7-source data 3.** Groupings of countries by geographical region. | |
| --- | --- |
| **Country** | **area_code** |
| Algeria | arab |
| Egypt | arab |
| Jordan | arab |
| Kuwait | arab |
| Lebanon | arab |
| Morocco | arab |
| Oman | arab |
| Qatar | arab |
| Saudi Arabia | arab |
| Syria | arab |
| Tunisia | arab |
| United Arab Emirates | arab |
| Georgia | commonwealth_is |
| Kazakhstan | commonwealth_is |
| Kyrgyzstan | commonwealth_is |
| Russia | commonwealth_is |
| Tajikistan | commonwealth_is |
| Uzbekistan | commonwealth_is |
| Brunei | e_asia |
| Cambodia | e_asia |
| China | e_asia |
| Indonesia | e_asia |
| Japan | e_asia |
| Malaysia | e_asia |
| Mongolia | e_asia |
| Myanmar [Burma] | e_asia |
| Philippines | e_asia |
| Singapore | e_asia |
| South Korea | e_asia |
| Taiwan | e_asia |
| Thailand | e_asia |
| Vietnam | e_asia |
| Argentina | lat_am |
| Bahamas | lat_am |
| Bolivia | lat_am |
| Brazil | lat_am |
| Chile | lat_am |
| Colombia | lat_am |
| Costa Rica | lat_am |
| Cuba | lat_am |
| Dominican Republic | lat_am |
| Ecuador | lat_am |
| El Salvador | lat_am |
| French Guiana | lat_am |
| Guyana | lat_am |
| Haiti | lat_am |
| Honduras | lat_am |
| Jamaica | lat_am |
| Mexico | lat_am |
| Nicaragua | lat_am |
| Panama | lat_am |
| Paraguay | lat_am |
| Peru | lat_am |
| Puerto Rico | lat_am |
| Trinidad and Tobago | lat_am |
| Uruguay | lat_am |
| Venezuela | lat_am |
| Canada | north_am |
| United States | north_am |
| Australia | oceania |
| Fiji | oceania |
| New Zealand | oceania |
| Albania | sce_europe |
| Bosnia and Herzegovina | sce_europe |
| Bulgaria | sce_europe |
| Croatia | sce_europe |
| Czech Republic | sce_europe |
| Estonia | sce_europe |
| Hungary | sce_europe |
| Latvia | sce_europe |
| Lithuania | sce_europe |
| Macedonia | sce_europe |
| Poland | sce_europe |
| Romania | sce_europe |
| Serbia | sce_europe |
| Slovakia | sce_europe |
| Slovenia | sce_europe |
| Turkey | sce_europe |
| Ukraine | sce_europe |
| Benin | ss_africa |
| Botswana | ss_africa |
| Burkina Faso | ss_africa |
| Burundi | ss_africa |
| Côte d’Ivoire | ss_africa |
| Cameroon | ss_africa |
| Congo | ss_africa |
| Congo - Brazzaville | ss_africa |
| Ethiopia | ss_africa |
| Ghana | ss_africa |
| Kenya | ss_africa |
| Lesotho | ss_africa |
| Madagascar | ss_africa |
| Malawi | ss_africa |
| Mali | ss_africa |
| Mauritius | ss_africa |
| Mozambique | ss_africa |
| Namibia | ss_africa |
| Niger | ss_africa |
| Réunion | ss_africa |
| Rwanda | ss_africa |
| Senegal | ss_africa |
| Seychelles | ss_africa |
| Sierra Leone | ss_africa |
| South Africa | ss_africa |
| Swaziland | ss_africa |
| Tanzania | ss_africa |
| Togo | ss_africa |
| Uganda | ss_africa |
| Zambia | ss_africa |
| Zimbabwe | ss_africa |
| Afghanistan | sw_asia |
| Bangladesh | sw_asia |
| India | sw_asia |
| Iran | sw_asia |
| Nepal | sw_asia |
| Pakistan | sw_asia |
| Sri Lanka | sw_asia |
| Austria | w_europe |
| Belgium | w_europe |
| Denmark | w_europe |
| Finland | w_europe |
| France | w_europe |
| Germany | w_europe |
| Greece | w_europe |
| Iceland | w_europe |
| Ireland | w_europe |
| Israel | w_europe |
| Italy | w_europe |
| Luxembourg | w_europe |
| Malta | w_europe |
| Monaco | w_europe |
| Netherlands | w_europe |
| Norway | w_europe |
| Portugal | w_europe |
| Spain | w_europe |
| Sweden | w_europe |
| Switzerland | w_europe |
| United Kingdom | w_europe |
