# Supplementary material for: Gender variations in citation distributions in medicine are very small and due to self-citation and journal prestige
Source: eLife. 2019 Jul 15;8:e45374. doi: 10.7554/eLife.45374 (PMC6677534; doi:10.7554/eLife.45374)
Supplement: Supplementary file 1. [file elife-45374-supp1.docx]

**Literature review**

| **reference** | **Field** | **Study type** | **Nation** | **N** | **Period** | **Data** | **Indicator** | **Statistical approach** | **Key result** |
| --- | --- | --- | --- | --- | --- | --- | --- | --- | --- |
| (Böhm et al., 2015) | Cardiology | Observation | GE | 1905 abstracts, 366 abstracts by women | 2006-2010 | Authors of abstracts submitted to the annual meetings of the German Cardiac Society | Journal impact factor | Mann–Whitney-U-Test. Only mean values are reported as results. | On average, women had published in journals with higher impact factor scores than men (women: 5.1 ± 0.2, Men: 4.4 ±, p= 0.000). |
| (Choi et al., 2009) | Radiation oncology | Observation | US | 826 authors, 234 women | 1997-2007 | Faculty at 78 US Radiation oncology departments | H-index. | Not specified | When stratified by academic rank no notable differences were found between women’s and men’s H-indices at the assistant professor, associate professor and full professor level. On average, women department chairs had lower H-indices than men department chairs. Results for full sample, men: N=592, Mean= 9.4 (95% CI: 8.7-10.01), women: N=234, Mean= 6.4 (95% CI: 5.5-7.4). Results for assistant professors, men: N= 188, Mean= 4 (95% CI: 3.4-4.6), women: N= 102 Mean= 4 (95% CI: 3.0-4.9). Results for associate professors, men: N= 131, Mean= 9.7 (95% CI: 8.6-10.8), women: N=46, Mean= 8 (95% CI: 6.2-9.8) Results for full professors, men: N=94, Mean= 17 (95% CI: 14.7-18.8), women: N=23, Mean= 17 (95% CI: 12.3-20.1). Results for department chairs, men: N=68, Mean= 18 (95% CI: 15.7-20.8), women: N= 11, Mean= 16 (95% CI: 12.7-19.8). |
| (Eloy et al., 2013) | 34 specialties | Observation | US | 9,952 authors, 3,133 women | 2012 | 25 institutions from the AMA’s Fellowship and Residency Electronic | H-index | Mann-Whitney U-test and Kruskall -Wallis Rank sum test. Only mean values are reported as results. | Women had lower average H-indices at all academic ranks from assistant professor level to chair/chief level. Interactive database. Results for full sample, women: N=3133, Mean= 5.59, men: N=6819, Mean= 10.25, p<0.0001. Results for assistant professors, women: N=1882, Mean= 3.77, men: N=2650, Mean= 2.60, p<0.0005. Results for associate professors: women: N=721, Mean= 7.14, men: N=1525, Mean= 8.76, p<0.0005. Results for full professors, women: N= 430, Mean= 14.65, men: N=2057 Mean= 17.22, p<0.0005. Results for department chairs, women: N= 100, Mean= 11.72, men: N=587, Mean= 18.98, p<0.0005. |
| (Frandsen et al., 2015) | Clinical research | Observation | DK | 134 researchers, 73 women | Five year period | PhDs enrolled at the Institute of Clinical Research, University of Southern Denmark. | Cumulative citation impact | Student’s t-test | In a comparison of male and female PhDs matched on sub-discipline, education, age and enrollment year, no notable average gender difference was found with respect to citation impact (women: Mean= 99.11, men: Mean= 105.95, p=0.798). |
| (Holliday et al., 2014) | Radiation oncology | Observation | US | 1,031 authors, 293 women | 1996-2012 | Faculty at 82 US academic radiation oncology departments | H-index and m-index | Mann-Whitney-U test | On average, women had slightly lower median m-indices than men (women: 0.47, men: 0.58, p<.05). On average, women had lower H-indices than men (women: 5, men: 8, p<.05). When stratified by rank, average differences in H-indices in favor of men were shown for all ranks with the exception of the assistant professor level. When stratified by rank, no statistically significant gender differences were shown for the m-quotient. |
| (Housri et al., 2008) | Academic surgery | Observation | GE | 994 abstracts, 96 with women authors | 2000-2004 | Authors of abstracts presented at the annual meetings of the German Association for Academic Surgery (GAAS) and the Society of University Surgeons (SUS) | Citation-rates per paper and journal impact factor | Student’s t-test | Results for SUS (N= 37 women and 300 men): No notable gender differences were observed in citation-rates per paper (women: 12.10 ± 4.47, men: 9.48 ± 0.60, p= 0.255). Gender differences in average. Journal impact factors were Gender differences in Journal Impact Factor scores were statistically insignificant (women: 3.27 ± 0.43, men: 2.67 ± 0.1, p= 0.063). The inconsequential results may be due to the small number of women included in the comparisons. Results for GAAS (N= 59 women and 590 men): Gender differences in average citation-rates per paper were statistically insignificant (women: 5.80 ± 0.98, men: 4.910 ± 0.35, p= 0.389). statistically insignificant (women: 4.741 ± 0.99, men: 3.348 ± 0.14, p= 0.063). |
| (Ingram, 2015) | Pediatric pulmonology | Observation | US | 85 authors, 35 women | 2014-2015 | 10 top-ranked departments in Pediatric pulmonology | H-index and m-quotient | Kruskal–Wallis rank sum test | Women had notably lower median H-indices than men (women: 3, men: 11, p= 0.002). Median-based gender differences for m-quotients were smaller and statistically insignificant (women: 0.41, men: 0.57, p=.09). This inconsequential result may be due to the samples used in the comparisons. |
| (Klimo et al., 2014) | Neurosurgery | Observation | US/CA | 312 authors, 52 women | 2008-2013 | Database of all neurosurgeons in North America | H-index and m-quotient | Not specified | Women had lower average H-indices than men (women: Mean= 8, men: Mean= 14, p= 0.001). Women had slightly lower m-quotients than men (women: Mean= 0.66, men: Mean= 0.52, p: 0.013) |
| (Larivière et al., 2011) | Health (broad) | Observation | CA | 6,231 authors (also includes other fields) | 2000-2008 | University and Clinical Professors at the universities in Quebec | Specialty-normalized journal impact factors and citations per paper | Comparison of mean values | On average, women published in slightly less prestigious journals (women: 1.17, men: 1.27) and had lower citation rates per paper (women: 1.23, men: 1.47). |
| (Martinez et al., 2015) | Musculoskeletal tumor research | Observation | US | 505 authors, 28 women | 2013 | Members of the Musculoskeletal Tumor Society | H-index | Multiple regression analysis | In a regression analysis adjusting for academic rank and experience, author gender was a statistically insignificant (p = 0.48) predictor of the H-index (No beta coefficient provided). |
| (Mueller et al., 2016) | Surgery | Observation, cohort study | US | 978 faculty, 234 women | 1950-2009 | Full-time faculty members of surgery departments of three academic centers | H-index, Cumulative citations | Student’s T-test | At the assistant professor level women had lower average H-indices than men (women: Mean= 8.15 (SD=6.41), men: Mean= 11.42 (SD=7.93), p= 0.002). No statistically significant gender differences in H-indices were identified for associate and full professors (numerical results and stratified sample sizes not reported). Likewise, no statistically significant differences were detected in the cumulative citation impact of women and men across the three ranks (numerical results and stratified sample sizes not reported). The inconsequential results may be explained by the small samples employed in each of the sub-group analyses. |
| (Mirnezami et al., 2016) | Medical science | Observation | CA | 1270 (Gender composition not specified) | 2000-2012 | Database of university funding in Quebec, disambiguated by gender | Discipline-normalized citation rates per paper | Random effect 2SLS regressions | Adjusting for multiple covariates including Journal Impact Factor, age and research funding, no statistically significant gender difference was detected in average citation rates per paper. Main predictor (0=male, 1=female): β= 0.0095, p>0.05. |
| (Nielsen, 2016) | Medical sciences (broad) | Observation | DK | 1,714 authors, 568 women | 2009 | Medical researchers at Aarhus University. Data retrieved from Web of Science | Self-citations, field-normalized citations per paper, Source normalized impact per paper | Mann-Whitney U-test | Women accrued lower average field-normalized citations per paper than men (women: Median= 0.72, men: Median= 0.87, p= 0.015). Women had lower source normalized impact per paper than men (women: Median: 1.26, men: Median: 1.17, p =0.01 ) |
| (Okhovati et al., 2015)Okhovati et al. 2016 | Epidemiology | Observation | IR | 91 authors, 14 women | 2013 | Web of Science, Researchers in Iran | H-index, AR-index and G-index | Multivariate linear regressions | Adjusting for scientific age and rank, the main predictor Gender (0= women, 1= men) was found to be an insignificant predictor of H-index scores (β= 1.36, p= 0.17) AR-index (β= 2.35, p= 0.22) and G-index (β= 0.34, p= 0.27). The inconsequential results may be explained by extremely small samples employed in the analyses. |
| (Pagel and Hudetz, 2011) | Anesthesiology | Observation | US | 1630 authors, 510 women | 1996-2011 | Faculty members from 24 US academic anesthesiology departments | H-index and citations per paper | Mann-Whitney U-test | Women had lower average H-indices than men. Women and men had similar citation rates per paper (numerical results not reported). |
| (Pagel and Hudetz, 2015) | Anaesthesia Education | Observation | US | 397 authors, 82 women | 2014-2015 | Grant recipients of the Foundation for Anesthesia Education and Research (FAER) grant program since 1987 | Citation rates, H-index | Mann-Whitney U-test | Women had lower average citations rates per paper than men (women: Median=18, men: Median= 23, p= 0.039). Women had lower average cumulative citation performance than men (women: Median= 327, men: Median = 827, p = 0.000). Women had lower average H-indices than men (women: Median= 10, men: Median= 14, p= 0.002). |
| (Paik et al., 2014) | Plastic surgery | Observation | US | 505 authors, 79 women | 2012 | AMA’s Fellowship and Residency Interactive Database. | H-index | Student’s t-test | At the assistant and associate professor level, women had lower average H-indices than men (women: N= 67, Mean= 5.1, men: N=254, Mean= 6.4, p= 0.04). The number of women at the full professor and department chair level was too small for meaningful statistical comparison. |
| (Pashkova et al., 2013) | Anesthesiology | Observation | US | 645 authors, 198 women | 2012 | Faculty at 25 US anesthesiology departments | H-index | Mann-Whitney U-test | When stratified by academic, rank no discernable difference was found between women’s and men’s H-indices at the assistant professor and associate professor level. Male full professors had notably larger average H-indices than female full professors (no numerical specifications, results are only presented in figures) |
| (Raj et al., 2016) | Medical science | Observation, cohort study | US | 1244 authors | 1995-2012 | Medical faculty from 24 medical schools | H-index | Negative binomial regression models | In a regression adjusting for race/ethnicity, specialty, setting and years since first appointment, women’s average H-index relative to men’s was = 0.81 (95% CI =.73-.90), P<0.0001). |
| (Rana et al., 2013) | Radiation Oncology | Observation | US | 607 authors, 203 women | 1996-2012 | Domestic radiation oncology residency-training institutions | H-index | Simple comparison of mean and median values | Women’s average H-index was 2.1 (95% CI: 1.7–2.4) and men’s was 2.7 (95% CI: 2.4–3.1). |
| (Susarla et al., 2015) | Oral and Maxillofacial Surgeons | Observation | US | 325 authors, 38 women | ? | American Association of Oral and Maxillofacial Surgeons (AAOMS) database | H-index | Bivariate analysis (means and SD) | No notable gender differences were detected in average H-indices (Women: Mean= 6.6 ± 8.0; Men: Mean= 6.6 ± 7.6). |
| (Winnik et al., 2012) | Cardiovascular research | Observation | US | 590 authors, 96 women | 2006 | Abstracts submitted to the European Society of Cardiology Congress in 2006 | Papers cited more than 10 times within 2 years after publication | Logistic regression | Both the gender of first authors (Male N: 217, Female N: 71) and last authors (Male N: 259, Female N: 25) were found to be insignificant predictors of producing papers with +10 citations. First authors (male = 0, female=1): Odds ratio: 1.34 (95% CI=0.066-2.73). Last authors (male= 0, female=1): Odds ratio: 0.22 (95% CI: 0.003-1.66). These inconsequential result may be explained by the small sample of women included in the analyses. |

**Search strategy**

Databases: PubMed and Google Scholar

Years: 2006 through 2016.

Search terms (all fields):

("citation impact" OR "scientific impact" OR "scientific quality" OR "publication quality" OR "publication impact" OR "research impact" OR "citation performance" OR "citation rate*" OR "research performance" OR "scientific performance" OR "publication performance" OR citations) AND (Gender OR Sex) AND (health OR Medicine)

Inclusion criteria: +Quantitative study, + numerical specifications on gender analysis of scientific performance, citation-related indices

**References**

Böhm M, Papoutsis K, Gottwik M, Ukena C. 2015. Publication performance of women compared to men in German cardiology. *International Journal of Cardiology* **181**:267–269. doi:[10.1016/j.ijcard.2014.12.018](https://doi.org/10.1016/j.ijcard.2014.12.018)

Choi M, Fuller CD, Thomas CR. 2009. Estimation of Citation-Based Scholarly Activity Among Radiation Oncology Faculty at Domestic Residency-Training Institutions: 1996-2007. *International Journal of Radiation Oncology Biology Physics* **74**:172–178. doi:[10.1016/j.ijrobp.2008.07.030](https://doi.org/10.1016/j.ijrobp.2008.07.030)

Darmoni SJ, Névéol A, Renard J-M, Gehanno J-F, Soualmia LF, Dahamna B, Thirion B. 2006. A MEDLINE categorization algorithm. *BMC Medical Informatics and Decision Making* **6**:7–7. doi:[10.1186/1472-6947-6-7](https://doi.org/10.1186/1472-6947-6-7)

Eloy JA, Svider PF, Cherla DV, Diaz L, Kovalerchik O, Mauro KM, Baredes S, Chandrasekhar SS. 2013. Gender disparities in research productivity among 9952 academic physicians. *Laryngoscope* **123**:1865–1875. doi:[10.1002/lary.24039](https://doi.org/10.1002/lary.24039)

Frandsen TF, Jacobsen RH, Wallin JA, Brixen K, Ousager J. 2015. Gender differences in scientific performance: A bibliometric matching analysis of Danish health sciences graduates. *Journal of Informetrics* **9**:1007–1017. doi:[10.1016/j.joi.2015.09.006](https://doi.org/10.1016/j.joi.2015.09.006)

Gehanno J-F, Ladner J, Rollin L, Dahamna B, Darmoni SJ. 2011. How are the different specialties represented in the major journals in general medicine? *BMC Medical Informatics and Decision Making* **11**. doi:[10.1186/1472-6947-11-3](https://doi.org/10.1186/1472-6947-11-3)

Holliday EB, Jagsi R, Wilson LD, Choi M, Thomas CR, Fuller CD. 2014. Gender Differences in Publication Productivity, Academic Position, Career Duration, and Funding Among U.S. Academic Radiation Oncology Faculty. *Academic Medicine* **89**:767–773. doi:[10.1097/ACM.0000000000000229](https://doi.org/10.1097/ACM.0000000000000229)

Housri N, Cheung MC, Koniaris LG, Zimmers TA. 2008. Scientific Impact of Women in Academic Surgery. *Journal of Surgical Research* **148**:13–16. doi:[10.1016/j.jss.2008.02.015](https://doi.org/10.1016/j.jss.2008.02.015)

Ingram DG. 2015. Publication Measures of Scholarly Productivity: Norms for Academic Pediatric Pulmonologists. *Pediatric Allergy, Immunology, and Pulmonology* **28**:183–186. doi:[10.1089/ped.2015.0511](https://doi.org/10.1089/ped.2015.0511)

Klimo PJ, Venable GT, Khan NR, Taylor DR, Shepherd BA, Thompson CJ, Selden NR. 2014. Bibliometric evaluation of pediatric neurosurgery in North America. *Journal of neurosurgery Pediatrics* **14**:695–703. doi:[10.3171/2014.8.PEDS1488](https://doi.org/10.3171/2014.8.PEDS1488)

Larivière V, Vignola-Gagné E, Villeneuve C, Gélinas P, Gingras Y. 2011. Sex differences in research funding, productivity and impact: an analysis of Québec university professors. *Scientometrics* **87**:483–498. doi:[10.1007/s11192-011-0369-y](https://doi.org/10.1007/s11192-011-0369-y)

Martinez M, Lopez S, Beebe K. 2015. Gender Comparison of Scholarly Production in the Musculoskeletal Tumor Society Using the Hirsch Index. *Journal of Surgical Education* **72**:1172–1178. doi:[10.1016/j.jsurg.2015.06.020](https://doi.org/10.1016/j.jsurg.2015.06.020)

Mirnezami SR, Beaudry C, Larivière V. 2016. What determines researchers’ scientific impact? A case study of Quebec researchers. *Science and Public Policy* **43**:262–274. doi:[10.1093/scipol/scv038](https://doi.org/10.1093/scipol/scv038)

Mueller CM, Gaudilliere DK, Kin C, Menorca R, Girod S. 2016. Gender disparities in scholarly productivity of US academic surgeons. *Journal of Surgical Research* **203**:28–33. doi:[10.1016/j.jss.2016.03.060](https://doi.org/10.1016/j.jss.2016.03.060)

Nielsen MW. 2016. Gender inequality and research performance: moving beyond individual-meritocratic explanations of academic advancement. *Studies in Higher Education* **41**:2044–2060. doi:[10.1080/03075079.2015.1007945](https://doi.org/10.1080/03075079.2015.1007945)

Okhovati M, Bazrafshan A, Zare M, Moradzade M, Mokhtari AM. 2015. Research Performance Measures and the Moderating Role of Faculty Characteristics in Epidemiology. *Global Journal of Health Science* **8**:72. doi:[10.5539/gjhs.v8n5p72](https://doi.org/10.5539/gjhs.v8n5p72)

Pagel PS, Hudetz JA. 2015. Scholarly Productivity and National Institutes of Health Funding of Foundation for Anesthesia Education and Research Grant Recipients: Insights from a bibliometric analysis. *Anesthesiology* **123**:683–691.

Pagel PS, Hudetz JA. 2011. An analysis of scholarly productivity in United States academic anaesthesiologists by citation bibliometrics. *Anaesthesia* **66**:873–878. doi:[10.1111/j.1365-2044.2011.06860.x](https://doi.org/10.1111/j.1365-2044.2011.06860.x)

Paik AM, Mady LJ, Villanueva NL, Goljo E, Svider PF, Ciminello F, Eloy JA. 2014. Research productivity and gender disparities: A look at academic plastic surgery. *Journal of Surgical Education* **71**:593–600. doi:[10.1016/j.jsurg.2014.01.010](https://doi.org/10.1016/j.jsurg.2014.01.010)

Pashkova AA, Svider PF, Chang CY, Diaz L, Eloy JA, Eloy JD. 2013. Gender disparity among US anaesthesiologists: Are women underrepresented in academic ranks and scholarly productivity? *Acta Anaesthesiologica Scandinavica* **57**:1058–1064. doi:[10.1111/aas.12141](https://doi.org/10.1111/aas.12141)

Raj A, Carr PL, Kaplan SE, Terrin N, Breeze JL, Freund KM. 2016. Longitudinal analysis of gender differences in academic productivity among medical faculty across 24 medical schools in the United States. *Academic Medicine* **91**:1074–1079. doi:[10.1097/ACM.0000000000001251](https://doi.org/10.1097/ACM.0000000000001251)

Rana S, Holliday EB, Jagsi R, Wilson LD, Choi M, Thomas CR, Fuller CD. 2013. Scholastic activity among radiation oncology residents at us academic institutions: A benchmark analysis. *Journal of Cancer Education* **28**:541–546. doi:[10.1007/s13187-013-0500-2](https://doi.org/10.1007/s13187-013-0500-2)

Susarla SM, Swanson EW, Lopez J, Peacock ZS, Dodson TB. 2015. Does self-citation influence quantitative measures of research productivity among academic oral and maxillofacial surgeons? *Journal of Oral and Maxillofacial Surgery* **73**:1981e1–1981e7. doi:[10.1016/j.joms.2015.05.018](https://doi.org/10.1016/j.joms.2015.05.018)

Winnik S, Raptis DA, Walker JH, Hasun M, Speer T, Clavien PA, Komajda M, Bax JJ, Tendera M, Fox K, Van De Werf F, Mundow C, Lüscher TF, Ruschitzka F, Matter CM. 2012. From abstract to impact in cardiovascular research: Factors predicting publication and citation. *European Heart Journal* **33**:3034–3045. doi:[10.1093/eurheartj/ehs113](https://doi.org/10.1093/eurheartj/ehs113)
